# Is the presence of accessory mandibular canals associated with the dimensions of the mandibular canal?

**DOI:** 10.2340/aos.v84.43600

**Published:** 2025-05-13

**Authors:** Emre Sözen, Hasan Akpınar

**Affiliations:** a Department of Oral and Maxillofacial Radiology, Faculty of Dentistry, Afyonkarahisar Health Science University, Afyonkarahisar, Turkiye; b Department of Oral and Maxillofacial Surgery, Faculty of Dentistry, Afyonkarahisar Health Science University, Afyonkarahisar, Turkiye

**Keywords:** Accessory mandibular canal, mandibular canal variations, mandibular canal measurements, CBCT

## Abstract

**Aim:**

The aim of this study is to classify accessory mandibular canals (AMC) and investigate their association with the dimensions of the mandibular canal (MC) to enhance surgical planning and prevent complications in dental treatments.

**Methods:**

Cone-beam computed tomography (CBCT) images were evaluated. AMC’s frequency, length, and diameter, including dental, superior, inferior, forward-open, forward-closed, and retromolar types, were determined. Additionally, the dimensions of the MC, including its length and diameter, were measured, and the relationship between the AMC and MC was statistically analyzed. Descriptive statistics, chi-square test, independent samples *t*-test (for normally distributed data), Kruskal-Wallis test (for non-normally distributed data), and Tamhane post hoc test were used for statistical analysis of the data.

**Results:**

AMC was identified in 82 of the 222 hemimandibles examined (36.9%). It was found that the length and diameter of AMCs varied significantly depending on the AMC type (*p* = 0.000). The diameter of the dental type AMCs (mean 0.40 ± 0.22 mm) was considerably smaller than that of other AMC types (mean 0.86 ± 0.37 mm). The length and diameter of the MC were measured as 69.20 ± 5.10 mm and 2.96 ± 0.57 mm, respectively. While MC length was not found to influence the presence of AMC (*p* = 0.785), MC diameter was significantly associated with the occurrence of AMC (*p* = 0.000).

**Conclusions:**

AMC, which is critical for improving surgical planning and reducing the risk of complications in dental procedures, is not uncommon. Their presence should be carefully evaluated, particularly in cases where the MC has a larger diameter.

## Introduction

Anatomic variations observed in the oral region are of significant importance in diagnosis and treatment. A thorough understanding of physiological anatomy is essential for accurately identifying pathologies and distinguishing anatomical variations. Both soft and hard tissue variations can be encountered in the oral cavity. Fordyce granules, leukoedema, fissured tongue, geographic tongue, linea alba buccalis, melanotic macule, physiological pigmentations, and lingual varices are among the commonly observed soft tissue anatomical variations in the oral cavity [1].

Common oral hard tissue anatomical variations include accessory foramina in the mandible, such as the accessory mental, lingual, and retromolar foramina, which hold clinical significance during dental procedures; bifid and trifid mandibular canal (MC) variations, which can impact dental implant planning and surgical interventions; maxillary sinus variations, including Haller cells, Concha Bullosa, and accessory ostia, which may contribute to sinusitis and other maxillary pathologies; and neurovascular variations in the anterior palate, such as additional foramina and canals, which are crucial for surgical planning and the prevention of complications. Although these variations are typically asymptomatic, they can sometimes mimic pathological conditions. Therefore, clinicians must possess comprehensive knowledge of these anatomical structures to ensure accurate diagnosis and avoid unnecessary interventions. [2–5].

One of the anatomical variations, the MC is a bony channel within the mandible that houses the inferior alveolar nerve, artery, and vein. It extends from the mandibular foramen to the mental foramen, providing neural and vascular supply to the lower teeth, the jawbone, and the surrounding soft tissues [6, 7]. Various surgical procedures, such as impacted tooth extraction, implant placement, cyst and tumor operations, orthognathic, and reconstructive surgery are performed in the mandibular region where the MC is located [8]. In addition, root canal treatments, prosthetic rehabilitations, and orthodontic applications can also have an impact on the MC [9]. Therefore, the ideal visualization of the MC during diagnosis and treatment processes is crucial for preventing potential complications. Additionally, since the anatomical variations within the MC can affect the success of dental anesthesia, they may also influence the outcomes of dental procedures performed in distant areas [6].

The radiographic visualization of the MC can be achieved using various techniques, including panoramic radiography, periapical radiography, magnetic resonance imaging (MRI), computed tomography (CT), and cone-beam computed tomography (CBCT) [10, 11]. Although panoramic radiographs are often the preferred method before dental procedures, they have certain limitations in clearly depicting the anatomical course and length of the MC. Insufficient visualization of the upper border of the MC, along with issues such as magnification, distortion, and ghost images, are other areas where panoramic radiography falls short. Furthermore, the detection of cariations within the MC and the visualization of accessory canals often do not achieve the desired success [12, 13].

The limitations observed in panoramic radiographs can be addressed with the use of CBCT. CBCT provides high-resolution, three-dimensional images that enable precise localization and assessment of the course of the MC and its relationship with surrounding structures [8]. CBCT is highly effective in identifying anatomical variations within the MC, such as bifid and trifid canals, accessory mandibular canals (AMC), anterior loops, and accessory mental foramina, which are often not visualized in conventional panoramic radiographs [12]. This detailed imaging method is of great significance for dental applications, such as surgical procedures related to the mandible, endodontic treatments, and dental implant procedures, as it contributes to the prevention of potential complications associated with the inferior alveolar nerve [14].

When anatomical variations are not detected during surgical planning, complications such as traumatic neuroma, paresthesia, anesthesia failure, and significant hemorrhage may occur during the surgical procedure [15]. During root canal treatment, excessive preparation that establishes a relationship with accessory canals can lead to mechanical trauma due to the files used, as well as chemical traumac resulting from irrigation solutions. Similar complications may also occur during apical resections [16]. In orthodontic mini-screw applications performed in the buccal shelf region, the position of the MC is crucial, as potential anatomical variations should be considered, as they may lead to unexpected complications [17]. In cases where anatomical variations of the mandibular canal position the neurovascular bundle in a manner susceptible to mechanical pressure, the use of removable prosthetic appliances may result in pain and discomfort due to repeated loading on these sensitive structures. [18].

Due to the significant impact of MC variations on dental procedures, there has been a need for the classification of these variations. These classifications are generally based on the branching of the MC and the paths followed by these branches, assessed using CBCT images [19]. For this purpose, the classification of bifid MC proposed by Naitoh et al. is frequently used [20]. The aim of this study is to investigate the characteristics of the MC and its variations, which have clinical significance in terms of postoperative complications and iatrogenic injuries.

## Materials and methods

### Study design

**Figure 1:**
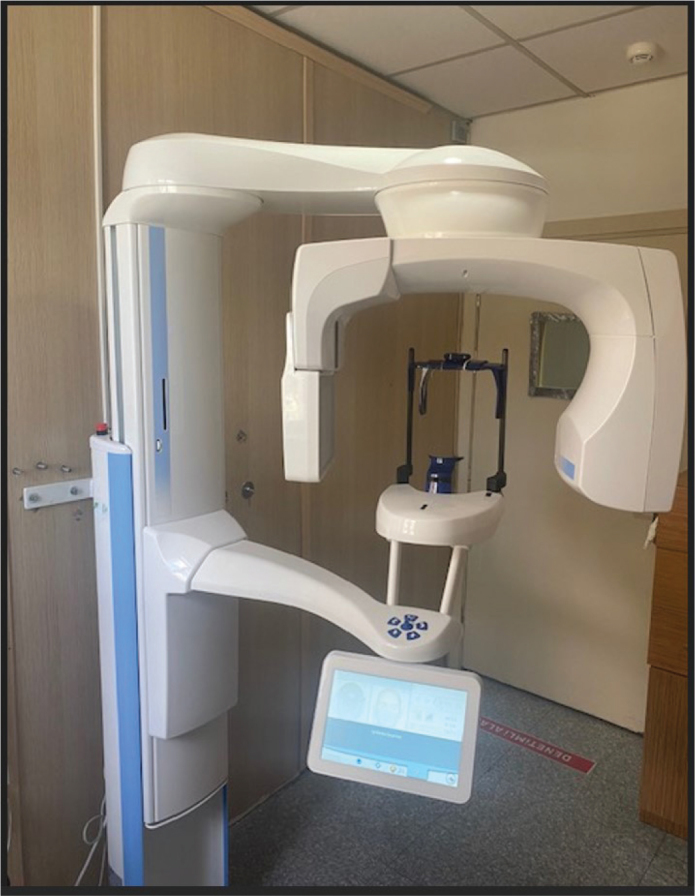
Cone-beam computed tomography device used for image acquisition (Planmeca ProMax 3D Mid).

This study was conducted in accordance with the Declaration of Helsinki. Ethical approval was obtained from the Clinical Research Ethics Committee of Afyonkarahisar Health Science University under the approval number 2021/4-259. CBCT images taken for various reasons at the Faculty of Dentistry, during the years 2022–2023 were included in the study. The Planmeca ProMax 3D Mid (Planmeca, Helsinki, Finland) device was used in our study (Figure 1). The imaging parameters were set to 90 kV, 8 mA, and a voxel size of 0.2 mm.

### Inclusion criteria

- Patients aged between 18 and 65 years.
- CBCT images obtained for any reason.
- Images suitable for bilateral evaluation of the MC.

### Exclusion criteria

- Artifacts such as motion or metal artifacts in the CBCT images that would hinder the evaluation of the MC.
- Large lesions involving the MC that obstruct its visualization.
- Presence of fractures in the mandible.

**Figure 2:**
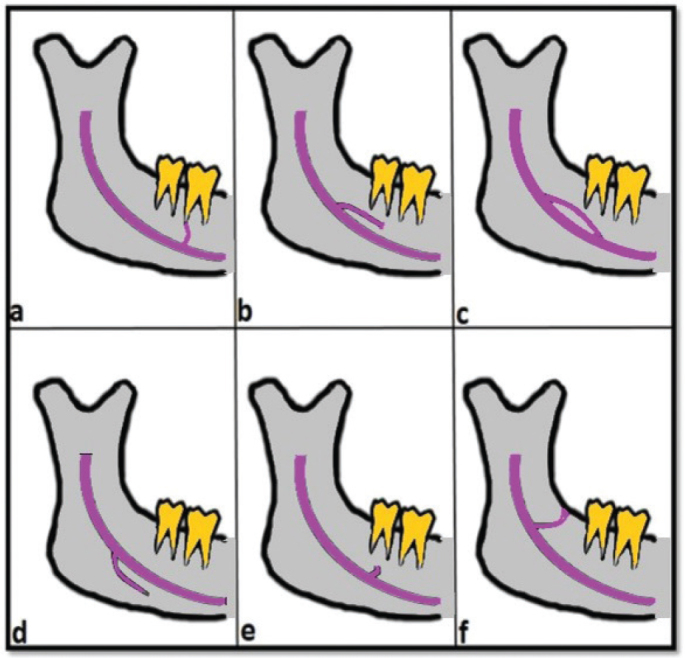
Classification of accessory mandibular canal types (A) Dental (B) Anterior open (C) Anterior closed (D) Inferior (E) Superior (F) Retromolar.

**Figure 3:**
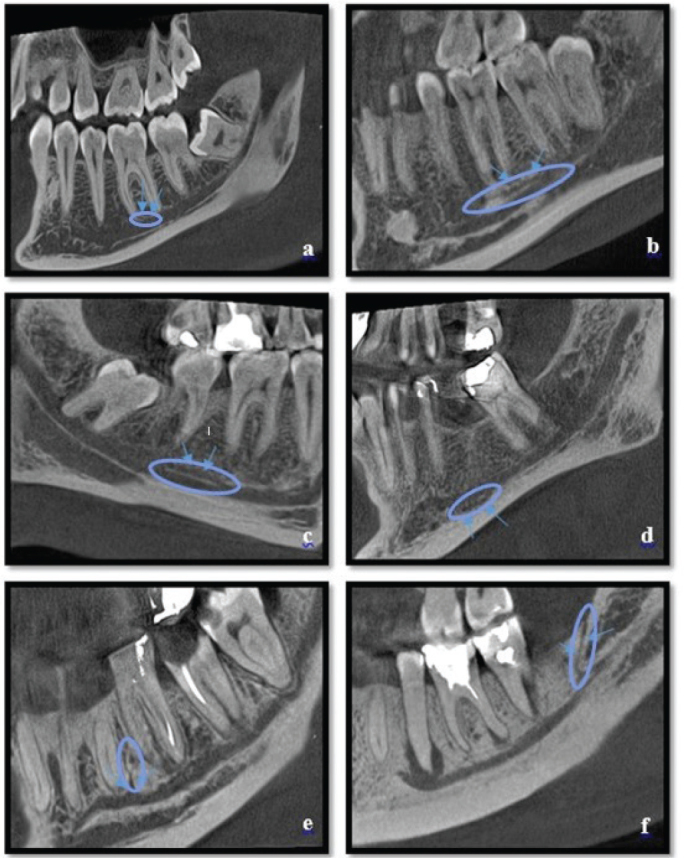
Variations of the mandibular canal: (A) Dental type (B) Anterior open type (C) Anterior closed type (D) Inferior type (E) Superior type (F) Retromolar type (Blue arrows indicate the variations).

For the measurement and analysis of CBCT images covering the bilateral mandibular molar and premolar regions, the software of the CBCT device was utilized. To measure the length and diameter of the MC, a panoramic image obtained from the CBCT passing through the MC was created. The widest canal diameter was measured and recorded on this image. For the measurement of MC length, the centerline of the canal was measured. The relationship between the MC length and diameter with canal variations was evaluated. The association of MC variations with age and gender was also assessed. The MC and its variations were examined and classified based on the images. This classification was carried out by modifying the classifications commonly used in the literature [20, 21]. MC variations and AMC types were divided into six classes (Figures 2 and 3). In our study, the classification used by Naitoh et al. was referenced and then revised for adaptation [22].

1. Dental type: The tip of the accessory branch extending from the main canal reaches the apices of molar or premolar teeth.
2. Anterior open type: The accessory branch extending from the upper wall of the main canal travels anteriorly but does not merge with the main canal.
3. Anterior closed type: The accessory branch extending from the upper wall of the main canal travels anteriorly and merges with the main canal.
4. Inferior type: The accessory branch extends inferiorly from the main canal.
5. Superior type: The accessory branch extends superiorly from the main canal.
6. Retromolar type: The accessory branch extending from the main canal reaches the retromolar foramen.

For the measurement of AMC length and diameter, a panoramic image derived from CBCT passing through the AMC was generated. In this image, the widest canal diameter was measured and recorded. For AMC length measurement, the length was determined by measuring along the axis that bisects the canal.

Cases in which dental-type AMC extended to different teeth or different roots of the same tooth within the same hemimandible were excluded from the classification. This approach was adopted to avoid potential complications in classification, as branching of the AMC to multiple teeth or multiple roots of a single tooth within the same hemimandible could render separate counting of each branch complex.

In our study, considering that some patients may have multiple types of AMC, these AMC types were planned to be treated as independent samples. The analysis of overall AMC prevalence and type-specific prevalence was conducted based on the presence or absence of variations.

### Statistical analysis

For all descriptive statistics and analyses, SPSS for Windows version 23.0 (SPSS Inc., Chicago, IL) was used. Initially, summary and descriptive statistics were employed for variables such as MC length and diameter, MC variations, age, and gender. The presence of MC variations was analyzed using the chi-square test about age groups and gender variables. The normality of the MC length and diameter variables was assessed using the Shapiro–Wilk test. Since the normal distribution was confirmed, the differences in MC length and diameter based on the presence of MC variations, gender, and age group were examined using an independent samples t-test. To evaluate the effect of AMC types on length and diameter, data normality was first assessed using the Shapiro–Wilk test. As normal distribution was not achieved, the Kruskal-Wallis test was applied. Given that the Kruskal-Wallis test indicated significant differences, the Tamhane test was subsequently used as a post hoc test. A significance level of 0.05 was set for all tests.

## Results

In our study, 222 CBCT images from 111 patients were analyzed. The age range of the patients was set between 18 and 65 years, with a mean age of 33.86 ± 13.35 years. Among the included patients, 43 were male and 68 were female.

### Occurrence rate of AMC

AMC was observed in 82 out of 222 hemimandible images (36.9%). To examine the effects of age group, gender, and side on the occurrence rates of AMC, a chi-square test was conducted, and no statistically significant differences were found. The occurrence rates of MC variations according to gender, age group, and side are presented in Table 1.

**Table 1:** Occurrence rates of mandibular canal variations according to gender, age group, and side.

Group	AMC % (*n*)	*p* *
***Gender***		
Male	38.4 (33)	0.726
Female	36.0 (49)	
***Side***		
Right	43.2 (48)	0.052
Left	30.6 (34)	
***Age group (years)***		
18–44	36.3 (58)	0.733
45–65	38.7 (24)	

AMC: accessory mandibular canals.

* *p* < 0.05

### Mean ages of patients with and without AMC

The mean age of patients without AMC was 33.59 ± 13.82 years, while the mean age of patients with AMC was 34.32 ± 12.59 years. According to the chi-square test results, no statistically significant difference was found between the mean ages of patients with and without AMC (*p* = 0.733).

### Distribution according to types of AMC

In 140 out of 222 hemimandibles, no AMC were observed, with the most frequently encountered AMC type being dental and the least frequently encountered type being inferior. Data regarding the frequency of AMC types are presented in Table 2. A single type of AMC was observed in 54 hemimandibles, while two different AMC types were noted in 25 hemimandibles, and three different AMC types were observed in 3 hemimandibles. In 38 different hemimandibles, dental-type AMC were observed, with 28 extending to a single tooth, 8 extending to two separate teeth, and 2 extending to three separate teeth. Additionally, of the 50 dental-type AMC extending to different teeth, 15 showed AMC extending to both the mesial and distal roots of the same tooth.

**Table 2:** Frequency of mandibular canal variations according to types.

AMC type	*n*	Frequency (%)*
No AMC	140	63.1
Dental	38	17.1
Superior	22	10
Retromolar	19	8.6
Anterior open	15	6.8
Anterior closed	13	5.9
Inferior	6	2.7

AMC: accessory mandibular canals.

* The total frequency appears higher due to the presence of multiple AMC types observed in the examined area.

#### Effect of AMC types on length and diameter

To assess the impact of AMC types on length and diameter, a normality test was initially conducted. Since the assumption of normality was not met, a nonparametric Kruskal-Wallis test was applied. Results from this test revealed that AMC types significantly affected length and diameter (*p* = 0.000). To identify the source of this effect, Tamhane’s Post Hoc test was performed separately, indicating that the diameter of the dental-type AMC differed significantly from other types. Regarding the impact of AMC types on canal length, no significant difference was found between dental and inferior types; however, these groups exhibited significant differences compared to other types (Table 3).

**Table 3:** The effect of accessory mandibular canal types on length and diameter.

AMC type	Diameter (Mean ± SD)	Length (Mean ± SD)
Dental	0.40 ± 0.22	5.06 ± 2.97
Superior	0.84 ± 0.4	11.7 ± 4.71
Anterior open	0.90 ± 0.28	11.71 ± 5.95
Anterior closed	0.81 ± 0.33	15.06 ± 7.89
Inferior	0.80 ± 0.28	7.04 ± 4.35
Retromolar	0.93 ± 0.47	12.7 ± 3.96

AMC: accessory mandibular canals.

#### The relationship between MC length and diameter with AMC

The normal distribution of the MC length and diameter variables was assessed using the Shapiro–Wilk test. Given that normal distribution was established, the differences in MC length and diameter based on the presence of AMC, gender, and age group were analyzed using an independent samples t-test. The MC lengths of all jaws included in the study were determined to be 69.2 ± 5.10 mm, while the MC diameter was found to be 2.96 ± 0.57 mm. The length and diameter data for jaws with and without AMC are presented in Table 4. It was observed that the MC length did not statistically significantly affect the presence of AMC (*p* = 0.785), whereas as the MC diameter increased, the occurrence of AMC significantly increased (*p* = 0.000). When examining the relationship between MC length and diameter and gender, it was noted that both the MC length and diameter were significantly greater in males (Table 5). When evaluating the relationship between MC length and diameter across age groups, no significant association was observed between age groups and the MC length or diameter (Table 6).

**Table 4:** The relationship between mandibular canal length and diameter with accessory mandibular canals.

Group	Mean ± SD (mm)	*p* *
**MC length**		
No AMC	69.27 ± 5.11	0.785
AMC	69.07 ± 5.11	
**MC diameter**		
No AMC	2.85 ± 0.51	0.000
AMC	3.14 ± 0.62	

AMC: accessory mandibular canals; MC: mandibular canal.

* *p* < 0.05

**Table 5:** The relationship between the length and diameter of the mandibular canal and gender.

Gender	Mean ± SD (mm)	*p* *
**MC length**		
Male (N:86)	71.72 ± 4.73	0.000
Female (N:136)	67.60 ± 4.67	
**MC diameter**		
Male (N:86)	3.17 ± 0.60	0.000
Female (N:136)	2.82 ± 0.51	

MC: mandibular canal.

* *p* < 0.05

**Table 6:** The relationship between mandibular canal length and diameter with age groups.

Age Group	Mean ± SD (mm)	*p* *
***MC Length***		
18–44 years	69.32 ± 4.96	0.536
45–65 years	68.86 ± 6.06	
***MC Diameter***		
18–44 years	2.89 ± 0.56	0.006
45–65 years	3.12 ± 0.56	

MC: mandibular canal.

* *p* < 0.05.

## Discussion

The anatomical variations of the MC and AMC are critical considerations before dental procedures. These canals can be observed at varying frequencies across different populations. Various methods have been employed in the literature to ascertain the prevalence of AMC. While cadaver studies have been reported for determining the prevalence of MC variations, the use of panoramic radiographs, CT, and CBCT imaging for this purpose is common [20, 23, 24]. Although CT imaging can be used for this purpose, its application in dentistry is quite limited due to cost, accessibility, and the risks associated with high radiation doses [25].

In the literature, the prevalence of the accessory canal has been found to vary between 10 and 65% across studies involving various ethnic populations [20, 26–31]. The considerable variation in the prevalence of the AMC can be attributed to a multitude of factors, including anatomical differences among individuals, ethnic diversity, the technical specifications of the imaging devices used, image resolution, and the methodological approaches adopted by researchers. Moreover, even among studies conducted within the same ethnic population, significant differences have been observed due to methodological variations. For instance, two separate studies conducted in Japan reported AMC prevalences of 15.5 and 65%, respectively, suggesting that the aforementioned factors may be responsible for these discrepancies [20, 31]. In the present study, the prevalence of AMC was determined to be 36.9%, whereas in different studies conducted in Türkiye, this rate ranges from 24.7 to 46.5% [12, 29, 32, 33].

Due to panoramic radiographs providing lower-resolution and two-dimensional images compared to CBCT, they are considered inadequate for detecting AMC, often reporting lower rates than actually present. Supporting this finding, a study in the literature utilizing panoramic radiographs reported an AMC prevalence of around 1% [34]. However, contrary to these findings, some studies indicate that anatomical structures, such as the mylohyoid nerve, may be misinterpreted as AMC due to erroneous evaluations, and that dense trabecular formations can mimic AMC. Consequently, this can lead to a reported prevalence of AMC that is higher than what actually exists. The literature emphasizes the significance of imaging techniques in accurately identifying these anatomical variations [35–37]. Due to the concerns mentioned in the literature regarding panoramic radiographs, CBCT has been preferred in our study.

In our study, the most frequently observed type of MC variation was the dental type (17.1%), which is consistent with the findings of Kuribayashi et al. [31]. However, in a similar population study by Orhan et al., the dental type was found to be at the lowest rate [29]. The lower prevalence of dental type AMC reported in other studies utilizing CBCT compared to the current study may suggest that this discrepancy is due to the smaller diameter of these types of AMC and the potential for very small-diameter dental type AMC to be overlooked [19, 38]. Additionally, a study conducted using MRI reported the presence of dental-type AMC extending to nearly all teeth [39]. The distinction of our study from the aforementioned studies can be attributed to the imaging method and the exclusion of dental type AMC that could not be clearly observed in the continuous CBCT sections.

However, the variability in the frequency distribution of variation types in different studies can be attributed to ethnic and methodological differences. For instance, studies conducted by Naitoh et al. [20] and Orhan et al. [29] reported the anterior type as the most common variation. These differences may vary particularly based on the classification systems used and the sample sizes in the studies. Haas et al. classified MC variations into retromolar and bifid MC, noting that the bifid MC is observed more frequently [3]. Due to the proximity of the retromolar foramen to the third molars, this variation should be considered during the extraction of impacted teeth. The prevalence of the retromolar foramen has been reported to vary between 3.2 and 26.58%, with differences noted in the diameter of the foramen and its distance from the third molars in the literature [40, 41].

Although all variations have distinct clinical significance, the retromandibular canal (RMC) is specifically examined in the literature. The neurovascular structures passing through the RMC provide innervation and blood supply to the mandibular molars, buccal region, posterior part of the alveolar ridge, and the mucosa overlying the retromolar fossa, while occasionally contributing additional vascularization to the temporalis and buccinator muscles. Therefore, understanding the anatomical variations of the retromolar is crucial for preserving neurovascular structures during surgical procedures [42]. However, the literature indicates that retromolar canals are not associated with neurosensory impairments following impacted third molar surgery [43].

The diameters and lengths of AMC are parameters that are frequently evaluated in the literature. In the current study, dental type AMC was identified as the narrowest variation, while the forward closed type was determined to be the longest variation. When evaluating the ratio of the diameter of the accessory canal to the diameter of the main canal, Rashsuren et al. [30] found this ratio to be approximately 0.5. In the present study, this ratio is at a level of 0.35. In the study by Zhang et al., while the length measurements of AMC are similar to those in our study, differences are observed in the diameter measure-ments [15]. It is suggested that the reason for this difference may be attributed to variations in the measurement locations for the diameters or ethnic differences [15, 30]. In contrast to the current study, although Kang et al. reported no significant differences in the diameters among different types of AMC, numerous studies have reported significant differences [15, 28, 44, 45]. When examining the length measurements of AMC, a wide range from 1.6 to 20 mm is observed in the literature. This broad range can be attributed to the use of different classifications of AMC. Indeed, the buccolingual type, reported as 1.6 mm, was not included in our study, aligning with many other studies in the literature [20, 30, 44, 45].

In the current study, the relationship between the diameter of the MC and the presence of AMC was examined, revealing that the incidence of AMC significantly increased with the enlargement of the MC diameter. This finding is consistent with results from the literature indicating that the diameter of the MC is associated with the presence of AMC [46, 47]. In our study, no significant relationship was found between the length of the MC and the incidence of AMC. Furthermore, there was no significant relationship observed between the presence of AMC and age or gender, which is consistent with the literature [45, 48]. In the study by Varvara et al., a distinction was made between right and left side measurements for the AMC, and the AMC was categorized into four types. While similarities exist between the two studies in terms of classification, our study reveals a higher average length for the retromolar canal and a lower average length for the dental canal [49].

In the present study, the relationship between the diameter and length of the MC and gender was also assessed. It was found that both the diameter and length of the MC were significantly greater in males than in females, which is consistent with the literature [50]. Additionally, our study demonstrated that an increase in the diameter of the MC may lead to a higher incidence of AMC. Although a higher proportion of AMC was observed in males, no statistically significant difference was found.

It has been reported that the inferior alveolar nerve is the most frequently injured nerve in the oral and maxillofacial region due to undetected AMC [19]. The literature reports that the development of paresthesia may occur due to damage to accessory canals following orthognathic surgery and apical resection procedures [51, 52]. It has also been reported that neurosensory changes may occur as a result of dental implants and impacted third molar surgeries; however, case reports indicate that symptoms regress upon removal of the causative factor [53, 54]. These accessory canals can adversely affect the success of dental anesthesia and may lead to significant complications during common surgical procedures, such as impacted third molar extractions. The literature includes a case where bleeding, which could not be controlled with local measures, was managed under general anesthesia using bipolar diathermy, bone wax packing, hemostatic cellulose, and sutures. This case revealed that the bleeding was caused by an accessory MC. This underscores the importance of identifying and considering AMC during surgical planning to mitigate potential complications [19, 55]. In cases involving the presence of AMC, intraoperative complications during implant surgeries and orthognathic procedures may include excessive bleeding, hematomas, and sensitivity changes, particularly in the retromolar region. Postoperative complications may include increased fibrous tissue formation related to implants, sensitivity disturbances, and elevated bleeding risks [56].

The presence of MC variations may increase the risk of complications in orthognathic surgeries. These variations, depending on the proximity of the MC to the cortical bone and its overall course, can not only alter the risk of complications but also impact treatment planning. Preoperative identification of these variations may necessitate modifications in the fixation technique and the type of osteotomy performed [57, 58].

In addition to the aforementioned complications, AMC may also contribute to the failure of dental anesthesia, which is a prerequisite for many treatments. In the clinical study conducted by Auluck et al., six cases of failed alveolar nerve blocks were examined in detail, and MC variations were identified in all cases [59].

## Limitations

The inclusion of a sample from a specific geographical region and a limited age range restricts the generalizability of the findings to a broader population. Additionally, as the evaluation of the obtained images relies on the observer’s interpretation, another limitation of this study is its dependence on subjective assessments. Furthermore, the relationship between the accessory MC and potential complications (e.g. paresthesia, bleeding) was not thoroughly investigated, which represents another constraint of this research. Future studies should conduct multicenter research encompassing diverse ethnic groups and geographic regions to comprehensively assess the population-based distribution of AMC. Additionally, the impact of AMC on surgical planning, its association with complications, and the diagnostic accuracy of different imaging techniques should be investigated, while CBCT-detected variations should be validated through histological and micro-CT analyses.

## Conclusion

According to the results of our study, the presence of AMC is significantly associated with the diameter of the MC, but no effect of MC length on the presence of AMC was found. The study highlights that the presence of AMC may influence surgical planning and increase the risk of complications in dental treatments. To prevent these complications, the use of CBCT is crucial. The MC diameter, which can also be detected through panoramic radiographs, provides valuable information. Particularly in cases with a wider MC diameter, the presence of AMC should be carefully evaluated.
